# Out-of-hours primary care. Implications of organisation on costs

**DOI:** 10.1186/1471-2296-7-29

**Published:** 2006-05-04

**Authors:** Caro JT van Uden, Andre JHA Ament, Gemma BWE Voss, Geertjan Wesseling, Ron AG Winkens, Onno CP van Schayck, Harry FJM Crebolder

**Affiliations:** 1 Department of Integrated Care, University Hospital Maastricht, Research Institute Caphri, Maastricht, The Netherlands; 2 Department of General Practice, Research Institute Caphri, Maastricht University, Maastricht, The Netherlands; 3 Department of health organization policy and economics, Research Institute Caphri, Maastricht University, Maastricht, The Netherlands; 4 Department of Clinical Epidemiology and Medical Technology Assessment, University Hospital Maastricht, Maastricht, The Netherlands; 5 Department of Respiratory Diseases, University Hospital Maastricht, Maastricht, The Netherlands

## Abstract

**Background:**

To perform out-of-hours primary care, Dutch general practitioners (GPs) have organised themselves in large-scale GP cooperatives. Roughly, two models of out-of-hours care can be distinguished; GP cooperatives working separate from the hospital emergency department (ED) and GP cooperatives integrated with the hospital ED. Research has shown differences in care utilisation between these two models; a significant shift in the integrated model from utilisation of ED care to primary care. These differences may have implications on costs, however, until now this has not been investigated. This study was performed to provide insight in costs of these two different models of out-of-hours care.

**Methods:**

Annual reports of two GP cooperatives (one separate from and one integrated with a hospital emergency department) in 2003 were analysed on costs and use of out-of-hours care. Costs were calculated per capita. Comparisons were made between the two cooperatives. In addition, a comparison was made between the costs of the hospital ED of the integrated model before and after the set up of the GP cooperative were analysed.

**Results:**

Costs per capita of the GP cooperative in the integrated model were slightly higher than in the separate model (ε 11.47 and ε 10.54 respectively). Differences were mainly caused by personnel and other costs, including transportation, interest, cleaning, computers and overhead. Despite a significant reduction in patients utilising ED care as a result of the introduction of the GP cooperative integrated within the ED, the costs of the ED remained the same.

**Conclusion:**

The study results show that the costs of primary care appear to be more dependent on the size of the population the cooperative covers than on the way the GP cooperative is organised, i.e. separated versus integrated. In addition, despite the substantial reduction of patients, locating the GP cooperative at the same site as the ED was found to have little effect on costs of the ED. Sharing more facilities and personnel between the ED and the GP cooperative may improve cost-efficiency.

## Background

Within the last ten years, out-of-hours primary care in the Netherlands has been substantially reorganised. Formerly, general practitioners (GP) used to be organised in small groups of GPs in which they joined a rota system. Nowadays large-scaled GP cooperatives have been set up to provide out-of-hours primary care. The current organisation of out-of-hours primary care is very similar to that in the UK and Denmark [1,2]. Many aspects of out-of-hours primary care have already been investigated, however, little is known about costs of current out-of-hours care as organised in GP cooperatives.

The organisation of out-of-hours care in the Netherlands varies from region to region. This is mainly due to local preferences and the rapid development of GP cooperatives. The GP cooperatives vary with respect to size of the population, number of participating GPs, accessibility, and location close to or separate from a hospital emergency department (ED). In the Netherlands, there is currently debate on the position and the role of the GP during out-of-hours care, and how out-of-hours care should be organised. More specifically, the debate focuses on the cooperation and the positioning between GP cooperatives and EDs. The Dutch minister of Health has argued that GP cooperatives should seek close collaboration with hospital emergency departments, and favours an integrated model in which the GP cooperative and the ED work closely together at the same site [3]. Many GPs however, are still reluctant to go towards a closer relationship with the hospitals' EDs, mainly because they are afraid to loose their identity and autonomy as GP. Obviously, it is essential that this discussion is supported with objective data on differences (advantages or disadvantages) between the out-of-hours care models.

In a recent publication we showed that different models of out-of-hours care, i.e. integrated versus separated out-of-hours system, have different implications on the utilisation of out-of-hours primary care [4]. In the integrated system the GP cooperative is located at the site of the hospital emergency department (ED) and sees all non-referred patients who attend the out-of-hours care facility. This ensures that no self-referred patient can enter the ED without first having been seen by a GP of the GP cooperative. The separated system has a GP cooperative located away from the hospital ED, and patients can choose to attend the primary care facility or the hospital ED. We found that the integrated model has the potential to reduce the number of patients utilising hospital emergency care with approximately 50% [5]. As a consequence, substantially more patients are seen at the integrated GP cooperative compared to the separated GP cooperative [4,5].

In addition to information on utilisation of out-of-hours care, we have also investigated patient satisfaction in South of the Netherlands, covering a region with one integrated cooperative and four separated cooperatives [6]. Detailed analysis, which was published in an internal report[7], showed that there were only few differences between cooperatives and they were not directly related to the way out-of-hours care was organised in relation to the hospital ED. However, in their comments, some patients mentioned preferring the integrated cooperative, because primary and hospital emergency care facilities are available at the same site.

So far, little is known about the differences in costs between an integrated out-of-hours care model and a separated out-of-hours care model. One would expect that reallocating patients during out-of-hours from hospital emergency care to primary care facilities may have effect on the costs of the GP cooperatives and the hospital ED. It is expected that the reduction of patients using ED care may cause a reduction in costs of the ED. It is evident that information on costs is necessary to support the discussion on which out-of-hours care organisation should be given preference to. There are two studies from the UK that investigated costs of out-of-hours care, but neither had an integrated GP cooperative in their analyses[8,9]. Nevertheless, Brogan et al[8] also suggested that integrating different out-of-hours services may lead to lower costs.

The objectives of this study are to determine the costs of two differently organised GP cooperatives (integrated versus separated), and to determine the effect of setting up a GP cooperative integrated with the ED on these costs.

## Methods

To gain insight in costs of a separated GP cooperative and an integrated GP cooperative we studied two cooperatives in the Southern part of the Netherlands. As an example of a separated model we chose the GP cooperative in the Heerlen region, and as a representative of an integrated cooperative we studied the GP cooperative in Maastricht. Also, data on use of out-of-hours primary care was collected. Costing is conducted from the perspective of the health services and costs to patients are not included.

### Setting

#### The separated GP cooperative

The separated GP cooperative was set up in March 1999. It started with taking care of a population of approximately 100,000, but expanded in 2002 to a population of 285,000. In this region one hospital ED is open during out-of-hours. The distance from the Heerlen GP cooperative to this ED is approximately 5 km. At the moment about 120 GPs participate in the separated GP cooperative. The GP cooperative is open from 5 pm to 8 am on weekdays, and from 5 pm on Friday to 8 am on Monday. In the evenings the GP cooperative is staffed with five GPs, and during the night only two GPs are present. During daytime in the weekends, the GP cooperative is staffed with seven to eight GPs.

Patients are expected to make a phone call before attending the cooperative. This allows the GP cooperative to triage patients at urgency levels of their medical complaints in order to prioritise treatment. This process of telephone triage is performed by doctor's assistants. In the evenings there are five to six doctor's assistants at the cooperative, and during the night there are only two. During the day in the weekends, eight or nine doctor's assistants are present.

#### The integrated GP cooperative

The integrated GP cooperative was set up in January 2000. During the first year, this GP cooperative covered only the population of the city of Maastricht (approximately 120,000 inhabitants). In August 2001 the surrounding area of Maastricht joined the GP cooperative, increasing the coverage area to 190,000 inhabitants. Only one ED is open for this region which is located at the same site as the GP cooperative. All patients attending the integrated out-of-hours care facility without referral are first seen by a GP, who refers, if necessary, the patient to the ED. At the moment 83 GPs participate in the integrated GP cooperative. The GP cooperative is open from 5 pm to 8 am on weekdays, and from 5 pm on Friday to 8 am on Monday. In the evenings the GP cooperative is staffed with three GPs on weekdays and four on weekends. During the night only two GPs are present. In the daytime on Saturday and Sunday, the GP cooperative is staffed with four GPs.

In the integrated out-of-hours care model patients are also expected to make a phone call first before attending the cooperative. However, patients are also allowed to attend without an appointment, although this is not preferred and discouraged. At the integrated cooperative telephone triage is performed by doctor's assistants and medical students. In the evenings on weekdays four doctor's assistants or medical students are present. At night on weekdays and weekends there is just one person who performs telephone triage. The number of doctor's assistants and medical students that is present during the daytime and evening on Saturday and Sunday varies between five and six.

Both GP cooperatives have installed management with a director or coordinator. The management of the integrated GP cooperative operates independently of the hospital. Patients contacting these two GP cooperatives can receive three types of consultations; telephone self-care advice, consultation at the GP cooperative, or a home visit. If necessary, patients are referred to the ED. GPs who perform home visits have a car with chauffeur at their disposal. In a previous paper we published information on conditions seen at both cooperatives[4]. We found that there was not much difference in conditions seen at both cooperatives, with one exception. At the integrated GP cooperative significantly more patients are seen with musculoskeletal disorders. This was found to be directly related to integrating the GP cooperative with the ED.

### Costs of both GP cooperatives

Information on costs was gathered from the annual accounts of the year 2003 of the two GP cooperatives involved in this study. This means that we used figures of actual costs. Costs have been divided in five categories: personnel, GPs' salary, accommodation, coordination and organisation, and other costs (including transportation, interest, cleaning, computers, communication, and overhead). Total costs per capita were calculated by dividing the total sum of costs by the number of inhabitants in the GP cooperative's coverage area. This was also repeated for the five costs categories.

### Primary care utilisation during out-of-hours

Information on use of primary care during out-of-hours at both GP cooperatives was collected from the annual reports. Per type of consultation, i.e. telephone advice, consultation at the GP cooperative, or home visit, number of patient contacts were registered.

### Cost calculation of emergency department

To study the effect of an integrated GP cooperative on costs of an ED, we assessed the costs of the ED of the University Hospital Maastricht. For this matter, the annual accounts of 1999 and 2000 were used (a year before and a year after the reorganisation of out-of-hours primary care). We did not assess the costs of the ED in the Heerlen region. During the years this study was conducted, two of the three former EDs in this region were closed. This will have caused considerable bias, which would make it impossible to assess changes in costs related to the set up of the separated cooperative without the interference of the closing of EDs.

## Results

The total costs of out-of-hours primary care in the separated GP cooperative have been found to be € 3.0 million. In the integrated cooperative this was € 2.2 million. In the separated model the costs of out-of-hours primary care are € 10.54 per capita per year and in the integrated model € 11.47 per capita per year. This difference is mainly the result of a difference in costs of personnel and 'other' costs per capita (Table 1). In the integrated cooperative the costs for personnel are € 4.01 per capita, while in the separated cooperative these costs are € 3.60 per capita. The category 'other' (including transportation, interest, cleaning, computers, communication, and overhead) in the integrated cooperative costs € 2.10 per capita, and € 1.54 in the separated cooperative. The costs of the GP salary are practically the same for both cooperatives; € 4.42 per capita in the integrated model, and € 4.52 per capita in the separated model. In total, about 75% of the costs of both GP cooperatives are based on personnel (including GPs' salary).

**Table 1:** Annual costs of an integrated GP cooperative and a separated GP cooperative in 2003.

	Integrated model		Separated model	
	2003		2003	

	Total	Per capita *(n = 190,000)*	Total	Per capita *(n = 285,000)*

Personnel/management	€ 761,484	€ 4.01	€ 1,025,561	€ 3.60
GPs' salary	€ 840,740	€ 4.42	€ 1,287,311	€ 4.52
Accommodation	€ 105,893	€ 0.56	€ 129,011	€ 0.45
Coordination and organisation	€ 71,970	€ 0.38	€ 122,754	€ 0.43
Other	€ 399,480	€ 2.10	€ 440,250	€ 1.54

Total	€ 2,179,567	€ 11.47	€ 3,004,987	€ 10.54

In total, about fifty-five thousand patient contacts were registered in 2003 with the integrated GP cooperative. The separated GP cooperative registered approximately seventy-six thousand patient contacts during out-of-hours (Table 2). This implies that the integrated GP cooperative (289 contacts/1000 inhabitants/year) has about 8% more patient contacts compared with the separated GP cooperative (267 contacts/1000 inhabitants/year). Over 66% of all contacts with the integrated GP cooperative consist of patients attending the GP cooperative for a consultation. In the separated GP cooperative about half of all contacts consist of consultations at the GP cooperative. Relatively more patients receive telephone advice in the separated model (36%) than in the integrated model (24%). Approximately 10% of all patient contacts at the integrated cooperative were home visits, which was not very different from that of the separated cooperative, where about 12% of all contacts were home visits.

**Table 2:** Utilisation of out-of-hours primary care in 2003.

	Integrated GP cooperative		Separated GP cooperative	
	2003		2003	

	n (%)	n/1000/year*	n (%)	n/1000/year*

Telephone consultation	13187 (24.0%)	69	27399 (36.0%)	96
Consultation at GP cooperative	36438 (66.3%)	192	39207 (51.5%)	138
Home visit	5350 (9.7%)	28	9466 (12.4%)	33

Total	54975 (100%)	289	76072 (100%)	267

* Number of patient contacts per thousand inhabitants per year.

The total costs of the ED in the integrated system, before the GP cooperative was established, were € 3.6 million (Table 3). In the year after the integrated GP cooperative was set up, the costs were slightly reduced (minus € 16,582) but remained around the € 3.6 million. The reduction was mainly caused by a reduction in costs related to the use of medication, bandages, plaster casts, and splints. These costs decreased from € 200,675 to € 185,624.

**Table 3:** Costs of the hospital emergency department before and after the establishment of the integrated GP cooperative.

	**1999 (before)**	**2000 (after)**	**Difference**
Personnel^a^	€ 1,250,611	€ 1,250,611	€ 0
Administration	€ 25,996	€ 22,770	- € 3,226
Communication	€ 7,453	€ 8,405	+ € 952
Interior	€ 3,078	€ 2,408	- € 670
Medication, bandages, casts, etc	€ 200,675	€ 185,624	- € 15,051
Diagnostics	€ 725,135	€ 726,400	+ € 1,265
Overhead^b^	€ 1,380,774	€ 1,380,774	€ 0
Other	14,107	14,254	+ € 147

Total	€ 3,607,830	€ 3,591,247	- € 16,582

^a ^Staffing of the ED remained unchanged; therefore, costs have been kept the same.

^b ^Overhead costs have been kept the same

## Discussion

The results of this study show that the primary care cooperative integrated with the ED is slightly more expensive, but has relatively more patient contacts, compared with the GP cooperative separate from the hospital EDs. There was no substantial change in costs of the ED at the integrated system after the GP cooperative had been set up, mainly because the organisation of the ED had not been changed despite the reduction of patient contacts.

The main category of costs of the GP cooperatives is that of personnel (doctor's assistants, management, and GPs), which is responsible for over three quarters of all costs. These costs could be in some way dependent on the model of out-of-hours primary care; differences in organisation may have specific effects on utilisation of out-of-hours primary care. As a consequence, staffing of the cooperative may have to be adjusted resulting from different demands. However, the costs for GPs are the same for both cooperatives; even slightly higher in the separated system. In contrast, costs of personnel (management, administration, and doctor's assistance) have been found to be higher in the integrated GP cooperative. However, this is probably the result of the scale advantage of the separated cooperative; the region covered by the separated cooperative is much larger than that of the integrated cooperative. Because costs like management, administration, but also accommodation have a less strong relationship with the size of the area, as compared to staffing of doctor's assistants and GPs, they will be relatively lower in a cooperative covering a larger area. Therefore, it seems that costs of out-of-hours care are more dominated by the size of the population the GP cooperative covers than the organisational structure of out-of-hours care, i.e. integrated versus separated. In this study we found that approximately 8% more patients attended the integrated GP cooperative compared with the separated GP cooperative. In that case, it is reasonable to suggest that the higher expenses (9% higher) of the integrated cooperative are justified by the fact that at this GP cooperative generally more patients are seen.

With respect to the ED of the integrated model no changes occurred in costs after the GP cooperative had been set up. Unfortunately we have not been able to use costs of the EDs in the separated setting to compare with the potential change we analysed in the integrated ED setting. Nevertheless, the before – after analysis we used provided a good indication of whether costs of this ED have changed over time. Because staffing of the ED remained unchanged, despite the substantial decrease of number of patients that utilised hospital emergency care [5], the costs of this department did not change. Staffing of the ED before and after the establishment of the GP cooperative did not change due to hospital regulations that prescribe a sufficient staffing in case of major traumata. Although costs of the ED remained the same, the regional Health Insurance Fund has cut the hospital's annual budget with approximately € 1.36 million. This budget reduction was mainly based on the fact that fewer patients attended the ED after the GP cooperative had been set up. For every patient attending the ED, the costs of a so-called first administrative consult (FAC) are reimbursed by the Health Insurance Company. Because fewer patients attended the ED, fewer FACs could be reimbursed, and consequently the hospital's budget was reduced.

A short (unpublished) questionnaire that was held under ED staff during the second year the integrated model was functioning, showed that they found to have more time for patients with severe complaints. Whereas, in the former situation they also had to take care of all minor injuries, which are now taken care of by the GPs.

Considering the utilisation of primary care in both settings, it is reasonable to suggest that the integrated primary care GP cooperative is equally cost efficient as the separated GP cooperative. After all, the higher costs (9% more) of the integrated GP cooperative are compensated by the larger number of patients (8% more) utilising out-of-hours primary care at the integrated GP cooperative. However, the ED at the integrated system has become less cost efficient because they see fewer patients at the same costs. For that matter, it would seem wise no longer keeping staff, like nurses, doctor's assistants, and management, separated in an integrated model.

Obviously, preference with either one of these two organisational models for out-of-hours care should not only be based on costs. Patient satisfaction and preferences with either one of these two systems should also be accounted for, but also the opinions of GPs should be considered. GPs also have to be satisfied with the organisation of out-of-hours care. First, because quality of care may be reduced in case of dissatisfied staff [10]. But second, because GPs have a strong saying in how out-of-hours care should be organised. Now that we have some indication that costs of out-of-hours primary care are only moderately dependent on the organisational structure, one could say that patients and GPs opinions, and other quality of care aspects should prevail in the decision of out-of-hours care organisation of preference. Nevertheless, from a financial point of view, based on the savings that occurred because the hospital's budget was reduced with 1.36 million because of decreased utilisation of ED care, the integrated out-of-hours care system should be preferred.

## Conclusion

In conclusion, the costs of out-of-hours primary care appear to be more dependent on the size of the population the cooperatives cover than on the way the GP cooperative is organised, i.e. separated versus integrated. In addition, locating the GP cooperative at the same site as the ED was found to have little effect on costs of the ED. Nevertheless, savings have occurred at the side of the Health Insurance Funds, which may prove to be beneficial to the community.

## Competing interests

The author(s) declare that they have no competing interests.

## Authors' contributions

CU participated in the design of the study, performed the statistical analysis, and drafted the manuscript. AA, GV, GW, RW, CS, and HC participated in the design of the study, supervised the project, and provided critical edits to this manuscript.

**Figure 1:**
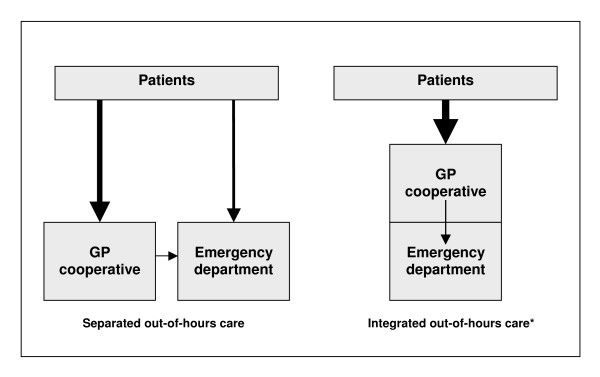
Organisation of out-of-hours care. * patients with referral or brought in by ambulance bypass this system and go directly to the ED.

## Pre-publication history

The pre-publication history for this paper can be accessed here:


